# *In Situ* Coherent X‑Ray Scattering Investigation of Macropore Formation in Porous Silica

**DOI:** 10.1021/acsomega.5c08905

**Published:** 2026-03-05

**Authors:** Lucas A. Portela, Aline R. Passos

**Affiliations:** Brazilian Center for Research in Energy and Materials (CNPEM), 42476 Brazilian Synchrotron Light Laboratory (LNLS), Campinas, São Paulo 13083-970, Brazil

## Abstract

Hierarchically porous silica was prepared via a sol–gel route accompanied by phase separation, using low-molecular-weight poly­(ethylene oxide) (PEO) as a phase separation inducer. The interplay between gelation and spinodal decomposition was investigated in situ through the combined use of ultra-small-angle X-ray scattering (USAXS) and X-ray photon correlation spectroscopy (XPCS), enabling simultaneous characterization of structural and dynamic evolution. The silica-rich domains are initially formed by ramified cluster aggregates, which progressively grow and reorganize into more compact structures as gelation proceeds. Arrest of the transient phase-separated state occurs when the gel network structure becomes established, dominated by superdiffusive dynamics, and constrains further growth of the separated domains. Increasing PEO content induces earlier gelation and earlier arrest of the phase-separated transient state, leading to thinner silica skeletons and smaller macropores in the final material. The results establish how the initial composition modulates the sol–gel transition and the arrest of separated domains, which control the pore size. The insights obtained in this work contribute to a deeper understanding of how gelation and phase separation govern the development of hierarchical porosity in silica materials, which is critical for designing materials with tailored structural and functional properties.

## Graphical abstract

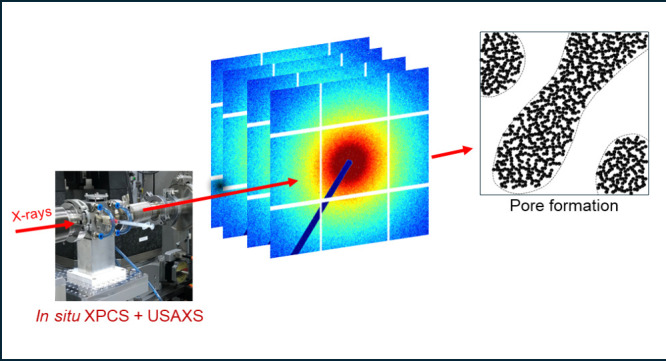

## 1 Introduction

Hierarchically porous materials exhibit two or more length scales and combine the benefits arising from each pore size regime.^1–6^ In fields involving liquid-phase reactions, hierarchically porous materials exhibit superior mass transport efficiency compared to nonporous or powdered counterparts.^7^ The interconnected macroporous network offers large diffusion pathways (pore diameter, *d*_*p*_ > 50 nm), while the mesopores (2 nm < *d*_*p*_ < 5 nm) contribute to a high surface area. Owing to their high surface area and efficient mass transport, these materials have been applied in the fields of liquid chromatography, adsorption, drug delivery, batteries, and catalysis.^3,7–9^

The strength of the sol–gel route for producing porous monoliths lies in its versatility, high degree of control over porosity, morphology, and chemical properties.^1,4,10,11^ The combination of spinodal decomposition by the addition of an organic polymer during the sol–gel transition provides an effective means of fabricating macroporous materials with interconnected pore structures. The progress of polycondensation reactions during gelation induces spinodal decomposition. This spontaneous process gives rise to a 3D interconnected two-phase morphology with controlled domain size distribution.^12,13^ The transient phase-separated structure is arrested by the sol–gel transition, and removal of the fluid-rich phase generates microscale pores. Mesopores are formed by the interstices in the network formed by the sol–gel transition. Moreover, additives can be incorporated into the gel-rich phase to enhance functionality for specific applications, such as the introduction of metal species for catalytic activity.^5,14^

Controlling gelation and phase separation simultaneously remains difficult as chemical bond formation dominates the gelation process. Precise control of the initial composition is required to induce phase separation during the sol–gel transition and to ensure that gelation occurs at the appropriate stage of phase separation. Furthermore, the evolution of the final morphology of the spinodally decomposed phase domains is predominantly controlled by dynamics governed by interfacial energy. In the late stages, coarsening driven by interfacial instability leads to the growth and stabilization of the domain structures, contributing to the development of the final pore structure. The influence of initial compositional parameters, including precursor, solvent, additives, and temperature, on the final porous morphology of silica systems has been extensively studied.^1,13,15–17^ However, there is a lack of studies demonstrating how these parameters affect the formation mechanism in real time through *in situ* characterization on the relevant macropore size scale. In addition, incorporating additives during the synthesis of macroporous materials can be an effective strategy for achieving highly dispersed systems within the macroporous framework. For catalytic applications, metal nanoparticles can be incorporated into porous silica. Among these metals, palladium (Pd) stands out due to its remarkable catalytic activity in a wide range of organic transformations, particularly in C–C bond formation and cross-coupling reactions such as Heck,^18^ Suzuki–Miyaura,^19^ and Sonogashira.^20^ A common approach to incorporate Pd species within the silica network is the cogelation method, in which the metal precursor is added to the sol before the gelation step.^21,14^ However, the sol–gel process coupled with phase separation is highly sensitive to the presence of additives, and the introduction of metal precursors can significantly alter the resulting pore size distribution and morphology.^6^ Nevertheless, it is crucial to understand how these additives affect the development of macroporous morphology.

The structural and dynamic evolution governing porosity formation during gelation and phase separation remains an experimental challenge. These processes typically initiate at the nanoscale, with domains progressively growing to microscopic dimensions over time. *In situ* small-angle X-ray scattering (SAXS) is an effective technique for investigating the formation of the first nanosized particles generated during hydrolysis and polycondensation and their subsequent aggregation into a gel network in TiO_2_,^22^ SiO_2_,^23^ and Al_2_O_3_ ^11,24^ systems. Similarly, *in situ* SAXS has also been used to probe the growth and aggregation of primary particles in porous alumina obtained through the sol–gel process combined with phase separation.^11,24^ The small structures formed during the early stages progressively evolve into features reaching hundreds of nanometers at intermediate and final stages, which can no longer be adequately resolved by SAXS. Furthermore, the underlying dynamics remain largely unknown, primarily due to the need to monitor a wide range of length and time scales simultaneously. A powerful strategy to overcome these challenges is the combined use of ultra-small-angle X-ray scattering (USAXS) and X-ray photon correlation spectroscopy (XPCS), which allows the simultaneous determination of structure and dynamics on length scales from nanometers to micrometers and on time scales from microseconds to minutes.^25–28^ XPCS is a coherent X-ray scattering technique that enables probing dynamics based on observations of fluctuations in the intensity of speckles.^29,30^ Simultaneously, USAXS information can be obtained via the ensemble-averaged scattering intensity, providing structural insights that enable real-time monitoring of hierarchical structure evolution within the same experiment. Recently, XPCS has contributed to addressing important questions related with gelation and phase separation in different soft matter systems such as phase separation in protein solutions,^31,32^ colloidal microscopic organization during gelation,^33^ structural evolution of thermoreversible gels,^34^ and relaxation in polymer electrolytes,^35^ among others. The complementary combination of USAXS and XPCS enables detailed insight into the structural and dynamic mechanisms driving gelation and phase separation, which is essential for the rational design of porous monolithic materials with tailored properties.

The main objective of this work is to unravel the gelation and phase separation mechanisms that control macropore formation in silica. By using tetraethyl orthosilicate (TEOS) and low-molecular-weight PEO as a phase separation inducer, we establish a model system that enables the formation of high-surface-area macroporous silica. The effect of palladium incorporation on the structure and formation pathway is also investigated. Through the combined application of *in situ* ultra-small-angle X-ray scattering (USAXS) and X-ray photon correlation spectroscopy (XPCS), we aim to track both the structural and dynamic evolution across multiple length and time scales. This approach allows us to elucidate the interplay between network formation and phase separation and to understand how coarsening controls the development of porosity at the late stage of phase separation. The insights obtained are expected to guide the design of porous materials with enhanced control over mass transport, promoting advances in areas such as catalysis and separation processes.

## 2 Experimental Section

### 2.1 Materials

Tetraethyl orthosilicate (TEOS, Sigma-Aldrich) and palladium chloride (PdCl_2_, Sigma-Aldrich) were used as the silica and Pd precursors, respectively. Poly­(ethylene oxide) (PEO, *M*_w_= 10000 g mol^–1^, Sigma-Aldrich) was utilized as a phase separation inducer. Nitric acid (HNO_3_, 65%, Labsynth) was used to catalyze TEOS hydrolysis and polycondensation. All reagents were used without further purification.

### 2.2 Synthesis

The silica gel synthesis was adapted from Nakanishi et al.^36^ using low-molecular-weight PEO. 0.17 g of PEO was dissolved in a mixture of 2.58 mL of deionized water and 0.19 mL of HNO_3_. The system was stirred for 30 min to ensure complete dissolution of PEO. Then, 2.13 mL of TEOS was added, and the solution was transferred to a 40 °C water bath, where it was maintained for 24 h to allow gelation and aging. The resulting gel was dried at 50 °C for 48 h. The sample was calcined in air to remove the PEO fraction. To prevent collapse of the silica structure during polymer removal, calcination was carried out in stages under conditions determined from thermal analysis (Figure S1). The dried gel was gradually heated to 190 °C and maintained at this temperature for 30 min. Then, the temperature was increased to 330 °C and held constant for 1 h. Finally, it was raised to 550 °C and maintained for 4 h. The molar composition was fixed as 4 TEOS: 60 H_2_O: 2 HNO_3_: *x* PEO, with *x* taking values of 0, 0.0063, and 0.0071 mol. The samples were named according to the PEO molar composition as Si0, SiPEO6, and SiPEO7, respectively. Palladium-silica samples were prepared by adding 0.0095 g of the metallic precursor to the initial water volume in the sample prepared with the molar composition: 4 TEOS: 60 H_2_O: 2 HNO_3_: 0.0071PEO and were named PdSiPEO7.

### 2.3 Characterization

The silica morphology was investigated by high-resolution scanning electronic microscopy (SEM, FEI, Inspect F50). The sample was dispersed in ethanol and dropped onto a copper grid. The microscope operated at 2 kV in scanning mode (SEM) and 30 kV in transmission mode (STEM). For pure silica, the STEM images were acquired in bright-field mode; for supported palladium, the images were acquired in dark-field mode to enhance the contrast between the nanoparticles and the support. Silica skeleton thickness and macropore diameter were statistically determined by measuring at least 100 uniform sections of silica skeletons and macropores in several selected STEM images (Figure S2). Nitrogen adsorption–desorption isotherms were obtained with a physisorption analyzer (Micromeritics, TriStar II 3020). The silica gels were pretreated for 12 h at 80 °C. The specific surface area was calculated by the Brunauer–Emmet–Teller (BET) method, and the pore size and pore volume was determined based on Barret–Joyner–Halenda (BJH) method. X-ray diffraction (XRD, Bruker, D8 Advance ECO) was used to identify crystalline structures. Cu Kα (λ = 1.54 Å) radiation was used as the incident beam. The 2θ was measured from 10° to 90° with a step size of 0.04°.

X-ray photon correlation spectroscopy (XPCS) and ultra-small-angle X-ray scattering (USAXS) experiments were performed at the Cateretê Beamline in the Brazilian National Synchrotron Light Laboratory using a coherent X-ray beam at 9 keV.^37^ The gelatinization process was investigated *in situ* at 40 °C over a period of 6 h. The initial suspension was freshly prepared and transferred into a capillary cell with a diameter of 1.5 mm using a syringe. The scattered X-rays were detected using a Pimega 540D detector positioned 20 m away from the samples, resulting in a q-range of 0.0002–0.047 Å^–1^, where q = 4πsinθ/λ, and λ is the X-ray wavelength (λ = 1.38 Å). A beam of ∼40 × 40 μm was focused on the sample. The incident flux of 2 × 10^10^ ph s^–1^ at 9 keV was attenuated using a sapphire absorber to minimize radiation damage, resulting in a final flux of 1.6 × 10^9^ ph s^–1^. Each XPCS acquisition lasted 500 s, with a 100 s interval before the next measurement. To prevent cumulative radiation damage, each XPCS series was collected at a fresh spot separated by 100 μm. Prior to the XPCS measurements, the radiation damage threshold was experimentally determined. This threshold was defined by the lack of detectable alterations in the USAXS profiles of a gel sample (Figure S3). USAXS data were obtained by azimuthal integration of the average scattering patterns. The resulting profiles were analyzed using the open-source scattering analysis software, SasView 5.0.6.^38^ The data were analyzed using the Guinier–Porod model^39^ to determine the radius of gyration (*R*_g_) and dimensionality of the scattering objects:

$$I(q)=\{\begin{array}{c} \frac{G}{q^{s}}\exp\left[\frac{-q^{2}R_{g}^{2}}{3-s}\right]\;\mathrm{for}\;q\leq q_{1}\\ \frac{D}{q^{\alpha }}\;\mathrm{for}\;q\geq q_{1} \end{array}$$ 1

where *q* is the scattering vector, *I*(*q*) is the scattered intensity, *R*_g_ is the radius of gyration, α is the Porod exponent, *G* and *D* are the Guinier and Porod scale factors, respectively, and s is the dimensionality parameter. For globular objects, such as spheres, *s* = 0; for rods, *s* = 1; and for lamellae, *s* = 2. The fit results are presented in Table S1 and Figure S4.

The XPCS autocorrelation function *g*_2_(*q*, *t*) was calculated according to (eq 2), with a Python software package available at the beamline.^40^

$$g_{2}\left(q,t\right)=\frac{\left\langle I\left(q,t_{0}\right)I\left(q,t_{0}+t\right)\right\rangle }{{\left\langle I\left(q,t\right)\right\rangle }^{2}}$$ 2

where *I*(*q*, *t*) is the scattered intensity measured at a scattering vector *q*, *t* is the lag time, and the bracket notation refers to time averaging over pixels in the corresponding *q* bin. The *g*_2_(*q*, *t*) function was modeled using the Kohlrausch–Williams–Watts (KWW) function (eq 3).

$$g_{2}\left(q,t\right)=A+{\beta e}^{-2{\left(t/\tau \left(q\right)\right)}^{\gamma \left(q\right)}}$$ 3

where *q* is the wavevector, *t* is the delay time, *A* is the baseline, β is the speckle contrast, τ is the relaxation time, and γ is the KWW exponent, which strongly depends on the nature of the underlying dynamics. The baseline was 1.004, and β was 0.1. The *g*_2_(*q*, *t*) was normalized according to (*g*_2_(*q*, *t*)-baseline)/β, such that the exponential decay starts at 1 and approaches a final baseline of 0.

The two-time correlation (TTC) was calculated according to (eq 4)­

$$C(q,t_{1},t_{2,})=\frac{\langle I(q,t_{1})I(q,t_{2})\rangle_{\mathrm{pix}}}{\langle I(q,t_{1})\rangle_{\mathrm{pix}}\langle I(q,t_{2})\rangle_{\mathrm{pix}}}$$ 4

where the bracket notation refers to averaging over detector pixels with the same *q* without any time averaging. TCC is typically represented as a 2D plot that correlates two patterns of series at times *t*_1_ and *t*_2_.

## 3 Results and Discussion

### 3.1 Morphological Control of Macrostructure

Macroporous silica with a controlled structure was synthesized via the sol–gel route combined with phase separation, using low-molecular-weight PEO as the phase separation inducer. Upon removal of the fluid phase during drying and elimination of organic compounds during calcination, the gel phase becomes the silica skeleton. When an appropriate amount of PEO is added to the sol–gel system, the phase separation process coincides with the sol–gel transition, resulting in a highly viscous, white, and opaque gel. In contrast, the sample synthesized without PEO (Si0) forms a highly viscous, transparent gel, indicating the absence of phase separation.

The morphology of the samples synthesized with varying amounts of PEO was investigated by SEM (Figure 1a–c), confirming the presence of highly interconnected macropores when an appropriate amount of PEO is used. Figure 1a reveals that, in the absence of PEO (Si0), silica exhibits a nonporous structure at the micrometer scale. In contrast, the SiPEO6 sample has a highly interconnected structure (Figure 1b). The macropore sizes range from approximately 100 nm to 4 μm, while the silica skeleton has a thickness between 600 nm and 3 μm (Figure S2). As the PEO content increases, the silica skeleton becomes thinner. In the SiPEO7 sample, the silica network exhibits more cracks, likely due to the increased fragility of the thinner skeleton combined with the larger amount of PEO to be removed during the calcination process. As a result, determining the skeleton thickness becomes more challenging. Nonetheless, a tendency toward thinner silica skeletons can be observed, with a size distribution from 100 nm to 3 μm (Figure S2). Therefore, both the silica structure and the macropore size depend on the PEO content.

**Figure 1:**
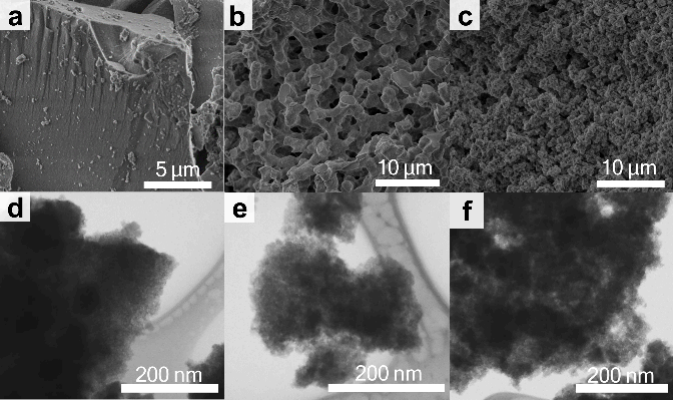
SEM images of the calcined samples prepared with different amounts of PEO, (a) Si0 (b) SiPEO6, (c) SiPEO7 and the corresponding STEM images in bright-field mode, (d) Si0, (e) SiPEO6, and (f) SiPEO7.

Further information about the morphology at the nanometric scale was obtained by high-resolution scanning electronic microscopy in transmission mode (STEM) (Figure 1d–f). Silica exhibits a highly ramified structure at the nanometer scale, regardless of the addition of PEO. The small voids observed in the silica structure result from the interconnected and ramified gel network formed during the polycondensation reaction. The intrinsic tendency of the sol–gel process to produce a branched network, due to hydrolyzed silica species forming multiple bonds and a three-dimensional arrangement, gives rise to mesopores in the final material.

### 3.2 *In Situ* USAXS and XPCS

USAXS provides access to length scales from tens to hundreds of nanometers, enabling detailed insight into the structural evolution of macropore formation. The structural evolution during the intermediate and late-stage gelation and spinodal decomposition was studied *in situ* using USAXS for samples without and with PEO (Figure 2). The gelation process progresses slowly, and during the early stages, the USAXS curves showed minimal changes, indicating that the initial silicate building blocks were not detectable within the investigated *q* range. After 220 min, a significant difference was observed between the samples prepared with and without PEO (Figure 2a,b). The Si0 sample is a homogeneous and nonmacroporous gel, and the USAXS curves are dominated by scattering of small silicate clusters, with the radius of gyration (*R*_g_) increasing from 6 to 9.4 nm (Figure 2e). This small increase in intensity and *R*_g_ suggests that the small clusters formed in the early stage of gelation grow slowly during the later stages. This result agrees well with the morphology observed in the calcined samples by STEM (Figure 1d), where the silica exhibits a highly ramified structure at the nanometric scale.

**Figure 2:**
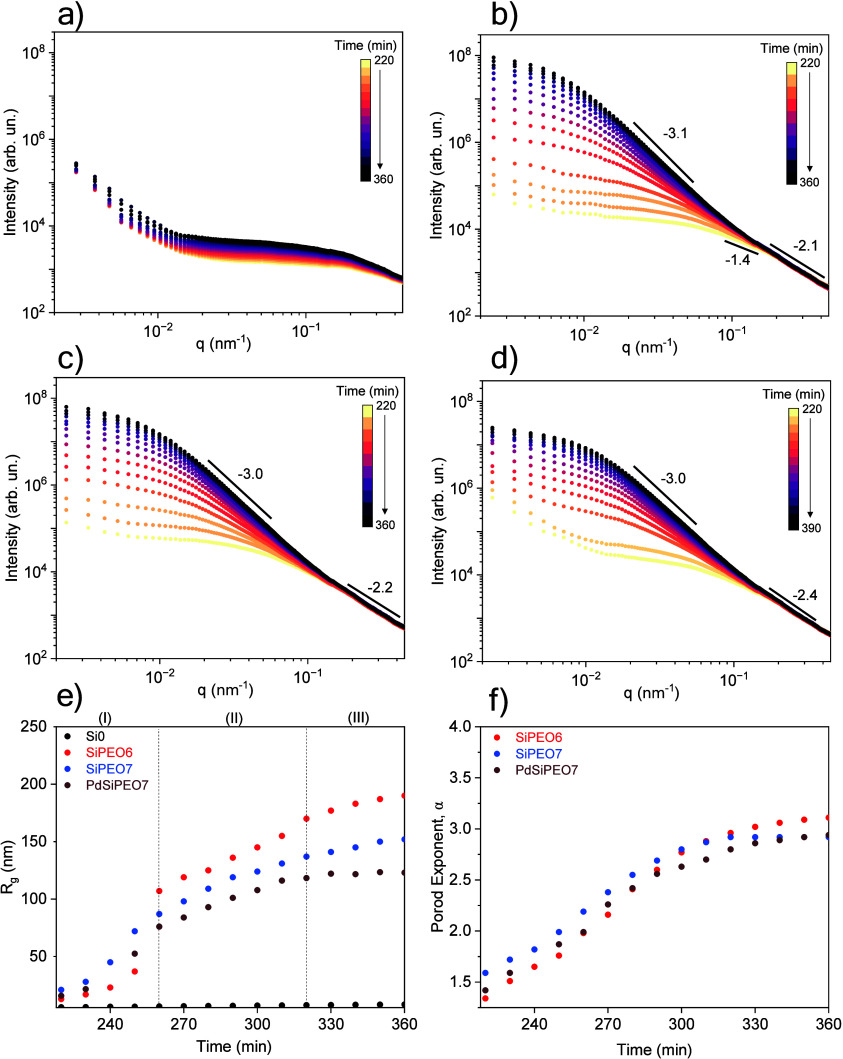
Temporal evolution of the USAXS profile during the gelation of (a) Si0, (b) SiPEO6, (c) SiPEO7, and (d) PdSiPEO7 and evolution of (e) radius of gyration (*R*_g_) and (f) Porod exponent for the corresponding samples.

In contrast, the SiPEO6 sample exhibits significant changes in the scattering profiles, and the intensity in the low-q region notably increases as gelation progresses (Figure 2b). The Guinier region shifts toward lower q values, accompanied by an increase in intensity with time, consistent with the growth of silica-rich domains resulting from phase separation. At 220 min, the increased *R*_g_ of 13 nm indicates the formation of large silica aggregates through cluster aggregation, likely driven by the local increase in silica species concentration within silica-rich domains. In the high-*q* region, the linear decay regime follows the Porod law (*I*(*q*) ∼ *q* ^–α^), with a Porod exponent α = 1.4, indicative of a ramified linear structure^41^ before evolving into more compact morphologies.

As gelation progresses in the SiPEO6 sample, the silica clusters grow and reorganize into denser aggregates in the silica-rich domains, exhibiting a Porod exponent of α = 3.3, which is characteristic of a rough surface (Figure 2f).^41^ The progressive increase in *R*_g_, reaching approximately 190 nm in the final stages, is consistent with the gradual growth of larger clusters in the separated silicate domains (Figure 2e). Although the determination of *R*_g_ at the later stages carries significant uncertainty due to the limited number of data points fulfilling the Guinier condition for globular objects (*qR*_g_ < 1.3), it is still possible to observe a consistent overall growth trend. In the final stage of spinodal decomposition, the initially branched linear structures and the silica-rich domains undergo coarsening, characterized by the slow growth of separated domains over time. To minimize free energy, the system reduces interfacial areas, resulting in domain growth and gradual interface smoothing. The presence of a rough interface may be attributed to the presence of bound PEO, resulting from hydrogen bonding between the ether groups of PEO and the silanol groups of silica. While this interaction has been previously suggested based on thermal analysis of the final decomposed phase in samples with high-molecular-weight PEO (*M*_w_ = 100 000 g mol^–1^),^15,42^ the use of *in situ* USAXS demonstrates that lower-molecular-weight PEO (*M*_w_ = 10000 g mol^–1^) adsorbs analogously on the surface of silica oligomers, forming a PEO- and silica-rich phase and a separate solvent-rich phase.

The deceleration in *R*_g_ growth after 260 min suggests the progressive development of a viscoelastic network as the polycondensation reaction reaches its late stages. During network formation, silica clusters grow progressively, and the emerging network restricts further coarsening of the phase-separated domains (Figure 2e). The elastic constraints imposed by the gel network can hinder domain mobility, resulting in a broad distribution of domain sizes. Similar results were observed for gluten protein gels, where anomalous liquid–liquid phase separation exhibited slow coarsening dynamics and broad size distributions, attributed to elastic constraints imposed by the gel network.^43^ Interestingly, the growth of silica species after 260 min separates into two regions: region (II) from 260 to 320 min, where a slowdown is observed, and region (III) >320 min, where the growth rate becomes even slower. This behavior likely reflects consolidation of the gel network, as detailed in the XPCS analysis of dynamic evolution.

The SiPEO7 sample with a higher amount of PEO exhibits behavior similar to that of SiPEO6, despite forming the gel network earlier (250 min) and smaller clusters at the end of gelation, with a maximum *R*_g_ of 152 nm (Figure 2e). After calcination, the silica skeleton is thinner than in the SiPEO6 sample, suggesting that the formation of smaller clusters during gelation may be associated with the development of smaller structures in the final material (Figure 1c). Therefore, earlier formation of the gel network arrests the transient state at an early stage of phase separation, resulting in smaller pores.

The addition of palladium does not inhibit the gelation process, and the scattering curves remain similar upon incorporation of palladium (Figure 2d). This indicates that palladium is dispersed as small particles within the silica matrix, and no evidence of large aggregates or phase-separated palladium domains is observed. The cluster size decreases with the addition of palladium, as indicated by the reduction in *R*_g_ (maximum 123 nm) in the sample PdSiPEO7 compared to the palladium-free SiPEO7 sample. In silica-metal systems, the influence of the metal on macropore morphology formation depends on the composition of the solvent-rich and silica-rich phases and the distribution of the organic polymer between these phases. In silica-nickel systems, when nickel nitrate is added during the hydrolysis of TEOS in the presence of PEO (*M*_w_ = 100 000 g mol^–1^), the PEO entraps the nickel species in the mesopores of the gel skeleton by forming coordination bonds between nickel cations and the ether oxygen of PEO.^14,44^ In silica-copper systems, copper cations can interact with the silica gel network, and no obvious interaction with PEO is observed.^44^ In this study, the addition of palladium has an effect similar to that of the addition of nickel in the system. The observed decrease in the silica-rich domain can be attributed to the interaction of palladium cations with PEO, which decreases the stability of the PEO complex with the silica gel network and leads to a decrease in the size of the silica domain.

The crystalline phase of palladium in the silica gel after drying and calcination was investigated by XRD. Figure 3a shows the XRD patterns of the calcined palladium-free SiPEO7 and the dried and calcined samples containing 1% Pd (PdSiPEO7). All samples exhibit a broad peak between 11 and 21 nm^–1^ attributed to amorphous silica. In the dried gel PdSiPEO7, no crystalline palladium phase peaks were observed, indicating the formation of small palladium particles. These results are consistent with the USAXS analysis, where during gelation, no evidence of large palladium aggregates was observed. After calcination, the PdSiPEO7 sample exhibits distinct peaks at 24, 37, 41, and 47 nm^–1^, which correspond to the (101), (112), (103), and (211) planes, respectively, assigned to the tetragonal PdO phase (JCPDS 41–1107). The appearance of these PdO peaks is attributed to the oxidation of palladium and partial particle growth during the calcination process.^21^ This aggregation suggests that Pd ions are more likely to interact with PEO rather than being incorporated into the silica network, as previously observed for nickel^14^ and copper^44^ ions during silica gel formation. Despite the aggregation after calcination, STEM images (Figure 3b) reveal a uniform distribution of palladium nanoparticles in the silica support. This uniform dispersion indicates that the addition of the palladium salt during the sol–gel process effectively promotes the formation of well-dispersed palladium nanoparticles within the silica network.

**Figure 3:**
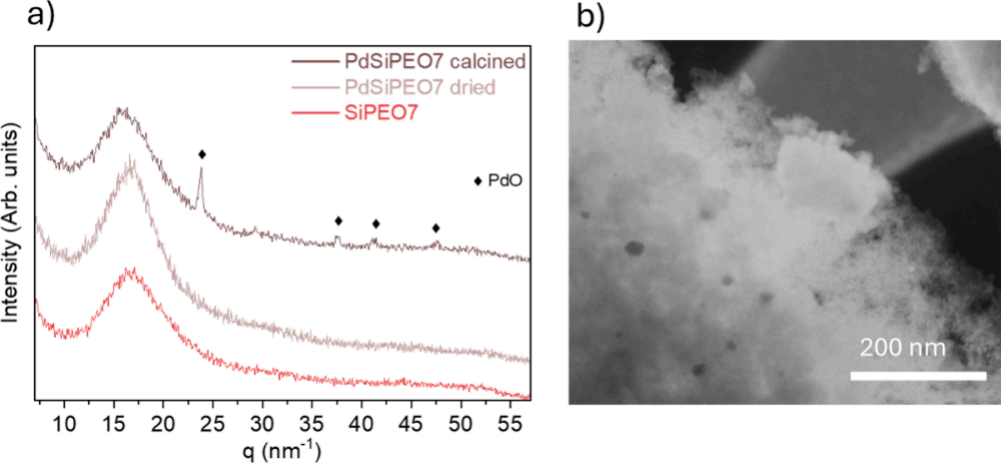
(a) XRD patterns and (b) STEM images in dark-field mode of calcined SiPEO7 and PdSiPEO7 after drying and calcination.

The evolution of collective dynamics in the silica-rich domains was investigated by XPCS measurements during late-stage gelation and phase separation. The gelation process progresses very slowly, and the dynamics were evaluated in time intervals in which no structural evolution was observed in the USAXS curves (Figure S3). Figure 4a–c shows the intensity autocorrelation functions (*g*_2_(*q*, *t*)) for the samples with PEO. In the absence of PEO, no decay in *g*_2_(*q*, *t*) was observed, which can be due to the low scattering intensity in the measured *q* range or to the presence of dynamics on time scales beyond the experimental window (Figure S5).

**Figure 4:**
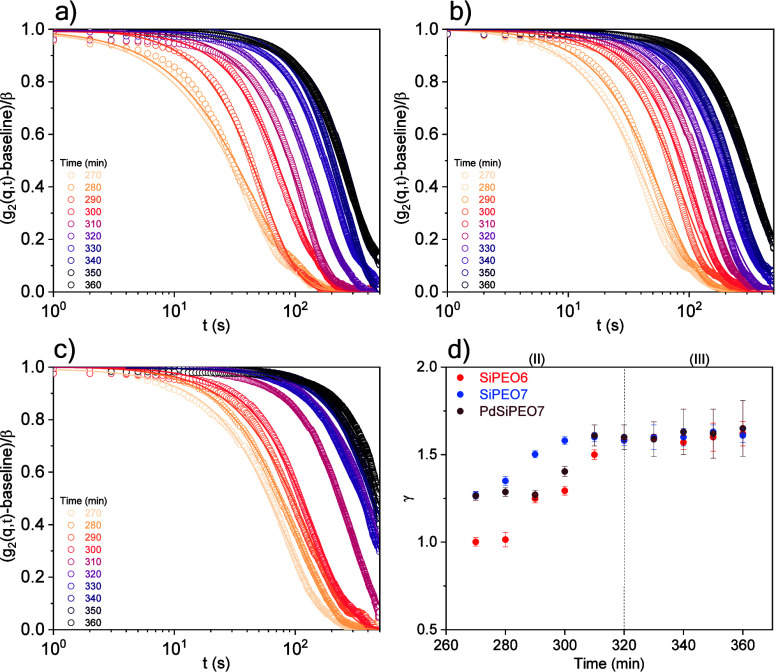
Normalized intensity autocorrelation functions, *g*_2_(*q*, *t*) at *q* = 0.0168 nm^–1^ for the samples (a) SiPEO6, (b) SiPEO7, and (c) PdSiPEO7; the error bars represent the standard deviations, and the solid curves represent the KWW fits. (d) Time evolution of the exponent γ.

For the samples with PEO, a decay in *g*_2_(*q*, *t*) becomes apparent after 270 min, corresponding to region II in the *R*_g_ evolution from USAXS, where silica clusters with characteristic sizes of hundreds of nanometers are observed. As gelation progresses, a systematic shift of the *g*_2_(*q*, *t*) decay to longer delay times is observed, indicating a tendency toward slower dynamics, which is associated with the increase in the size of silica clusters and the crowding of silica-rich domains. The dynamics are characterized by the KWW exponent γ (eq 3), where γ = 1 for simple diffusive Brownian motion, γ < 1 for subdiffusive dynamics, commonly observed in the liquid state near the glass transition,^45–47^ and γ > 1 for superdiffusive dynamics, often observed in disordered soft solids.^45,48,49^ The time evolution of γ is shown in Figure 4d. For the sample SiPEO6 at gelation times below 280 min, the *g*_2_(*q*, *t*) exhibits a single exponential shape (γ = 1), which is characteristic of Brownian motion in a simple liquid. The type of dynamics is further investigated by the *q*-dependence of the relaxation rate, Γ ∝ *q* ^*n*^, where the exponent *n* characterizes the nature of the dynamics. At 270 min, an exponent close to 2 indicates diffusive dynamics (Figure S6). The diffusive dynamics can be interpreted as the motion of silica clusters in the separated silica-rich domains. After 290 min, the dynamics become dominated by a compressed decay, indicating a transition in the nature of the dynamics. The compressed exponential shape (γ > 1) is attributed to superdiffusive dynamics, which have been reported as stress-dominated dynamics, commonly observed in gels,^48,50–53^ glass,^45,54,55^ and polymers.^49,56^ Such superdiffusive motion in jammed states is indicative of dynamic arrest, where fluctuations are constrained by structural limitations.^53^ Furthermore, the exponent extracted from the Γ ∝ *q* ^*n*^ dependence reaches values close to 1 after 290 min (Figure S6), which is characteristic of nondiffusive motion and is typically attributed to ballistic or superdiffusive dynamics.^46,50,51^ As gelation progresses, the continuous slowing down of the dynamics reflects strong suppression of network fluctuations. After 320 min (region III in the *R*_g_ evolution), γ reaches values around 1.6 and remains almost constant, consistent with dynamics that are largely unchanged once the gel network is formed. Similar behavior characterized by γ > 1 and *n* ≈ 1 has been reported in various gel and glassy systems, including glass-forming polymers,^49^ colloidal particles,^46^ epoxy silicate composites,^52^ and proteins.^51^ The onset of superdiffusive dynamics likely drives the arrest of the transient phase-separated structure and controls the macropore-size distribution in the final material. Similar superdiffusive behavior associated with the arrest of spinodal decomposition has been reported in thermoreversible colloidal systems with moderate-range attractions, evidenced by time-averaged microscopy (TAM) and differential dynamic microscopy (DDM).^57^ The gel is characterized by isotropic dynamics (Figure S8).

With increased PEO content in SiPEO7, superdiffusive dynamics are observed earlier (270 min), indicating an earlier onset of dynamical arrest. SEM confirms the formation of smaller macropores (Figure 1). The addition of palladium does not significantly affect the dynamics of the silica-rich domains, consistent with prior results showing that palladium does not bond to silica species. Although the dynamic regime is unchanged, the relaxation time after 310 min becomes longer, but this estimate carries uncertainty because the decay is not fully captured (Figure S7).

Out-of-equilibrium dynamics of the gel were further characterized by analysis of the two-time correlation function (TTC) (eq 4). Figure 5 shows the TCC for the sample SiPEO6 at different times. Warm colors indicate strong intensity–intensity correlation between two experimental times, whereas cold colors represent weak correlation. At 290 min, the dynamics are homogeneous, as indicated by a warm-colored stripe approximately parallel along the central diagonal. This feature indicates that the relaxation time remains constant over the experimentally accessible time window. After 330 min, broadening of the high-correlation region with increasing time reveals the time-dependent nature of the gel-network dynamics. This evolution reflects gel aging, with a gradual slowdown of the dynamics observed as the reaction time increases. The slowdown followed by a slight acceleration at 330 min likely corresponds to local rearrangements within the network after dynamic arrest, which temporarily modify the relaxation rate before complete stabilization. After this period, the consolidated gel network exhibits aging behavior.

**Figure 5:**
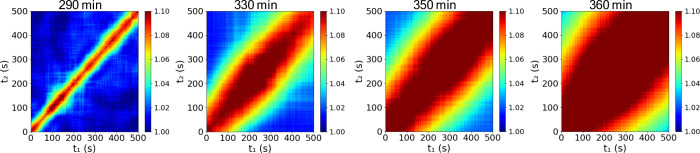
Two-time correlation function at *q* = 0.0168 nm^–1^ for the SiPEO6 sample at selected times.

Based on the USAXS and XPCS results, an idealized model was proposed to illustrate macropore formation in the SiPEO6 sample (Scheme 1). At 220 min, the silica-rich separated domains are formed by ramified cluster aggregates of approximately 13 nm. As gelation progresses, the clusters increase in size and the silica domains grow, forming more compact aggregates. After 260 min, the silica-rich domains continue to coarsen, slowly increasing in size and developing into more compact structures. The diffusive dynamics observed in the sample SiPEO6 reflect the diffusive motion of clusters in separated silicate domains, indicating a fluid state in which clusters later coarsen into larger entities. Arrest of the transient phase-separated state occurs around 290 min for the sample SiPEO6. At 320 min, the gel network structure becomes established and begins to age, constraining further growth of the separated domains by coarsening. Increasing the PEO content induces an earlier onset of dynamic arrest. The system progressively slows, and at late times the dynamics are largely unchanged once the gel network has formed. The evolution toward the arrested transient state resembles observations from simulation studies of colloidal and molecular fluids, where phase separation proceeds through coarsening of the dense domains.^58^

**Scheme 1:**
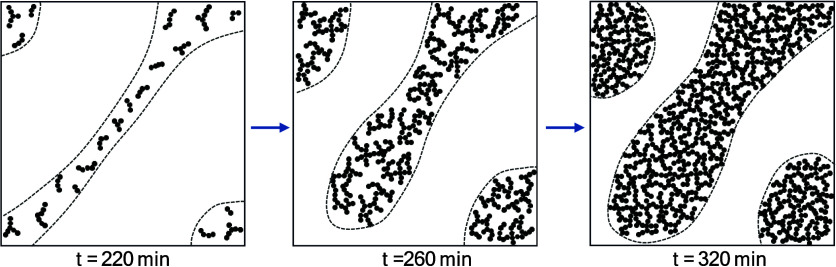
Illustration of Macropore Formation for the SiPEO6 Sample^a^

a The dashed line indicates the silica-rich domains undergoing phase separation.

The final mesoporous structure of the calcined samples was investigated by using nitrogen adsorption/desorption (Figure 6). The presence of mesopores in the sample without PEO is evidenced by a type IV adsorption isotherm, characterized by a hysteresis loop at intermediate relative pressures.^59^ The hysteresis loop exhibits an H2-type shape, which is characteristic of pores with an ink-bottle morphology. The small mesopores with an average diameter of approximately 3.4 nm are probably formed by the interstitial spaces between the silica gel networks, as observed by STEM (Figure 1d). As the PEO concentration increases, a slight increase in the average mesopore diameter is observed, ranging from 3.5 to 3.7, while the overall pore morphology remains unchanged. With increased PEO content, the SiPEO7 sample displays two distinct pore diameters, 3.7 and 5 nm, suggesting the development of a heterogeneous structure within the silica network. This could be attributed to crack formation during the removal of substantial amounts of PEO, as observed in the SEM images (Figure 1b). In addition, a change in pore morphology is observed, as indicated by a hysteresis loop displaying characteristics of both H2 and H3 types, suggesting the presence of complex pore structures, including ink-bottle-shaped pores and slit-like pores typically formed between particles.^59^ Table 1 presents the surface area calculated using the BET method and the mesopore diameter and volume determined by the BJH method. The samples exhibit a remarkably high specific surface area, reaching a maximum of 718 m^2^ g^–1^. The addition of PEO not only contributes to the formation of a macroporous structure but also influences mesopore size and volume as its concentration increases. Similar results were also observed for nickel^14,44^ and copper^44^ ions introduced during the sol–gel transition of silica. With the addition of palladium, the partial filling of small pores, measuring 5 nm in the corresponding sample without palladium (SiPEO7), suggests that the metal particles are partially distributed within the mesopores. As a result of this partial filling of the mesopores, a decrease in the surface area was observed.

**Figure 6:**
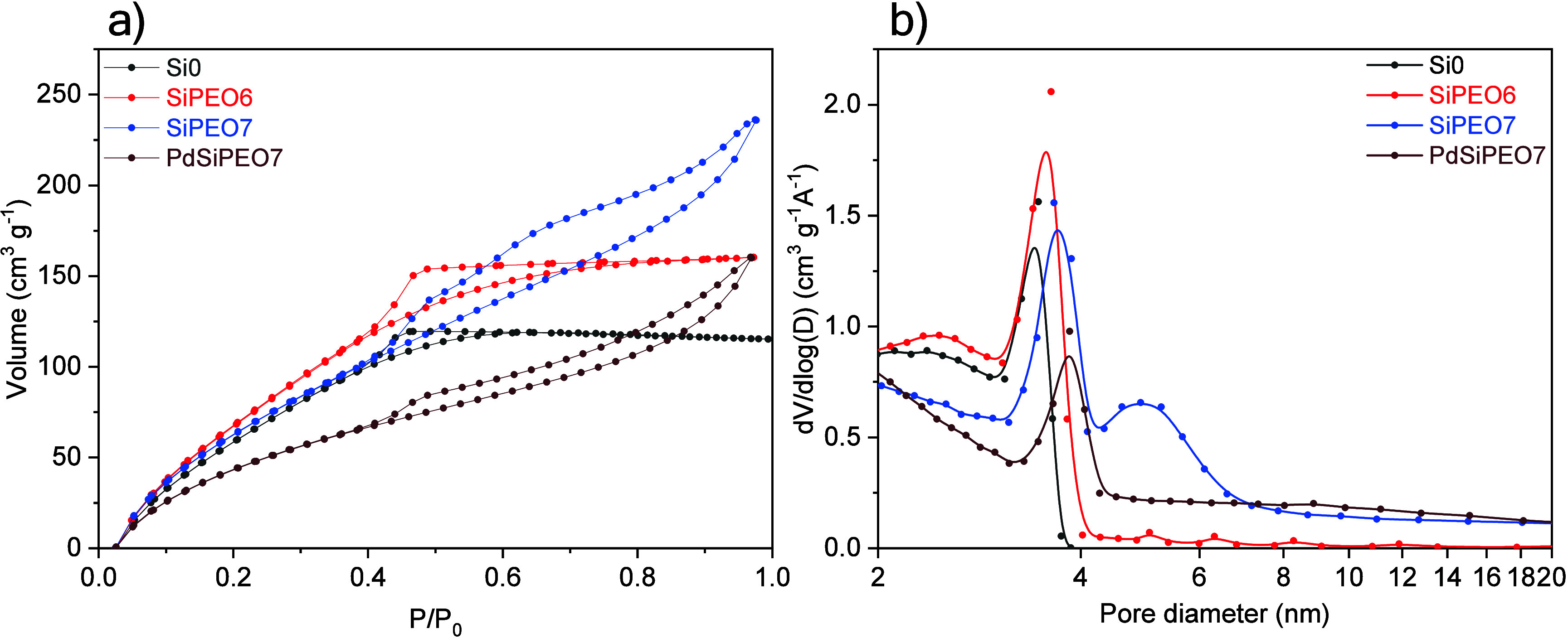
(a) Nitrogen adsorption–desorption isotherms and (b) pore size distribution of samples synthesized with different amounts of PEO and with palladium addition.

**Table 1:** BET Surface Area, Average Diameter, and Mesopore Volume of the Samples Synthesized with Varying Amounts of PEO

sample	S_BET_ (m^2^g^–1^)	D (nm)	V (cm^3^g^–1^)
Si0	623 ± 4	3.4 ± 0.3	0.3
SiPEO6	718 ± 5	3.5 ± 0.4	0.4
SiPEO7	690 ± 8	3.7 ± 0.5 and 5 ± 1	0.5
PdSiPEO7	533 ± 8	3.8 ± 0.4	0.4

## 4 Conclusions

We have investigated the structural and dynamic mechanisms underlying macropore formation in silica during the sol–gel transition accompanied by phase separation. The combination of *in situ* USAXS and XPCS reveals that silica-rich domains evolve from ramified aggregates into compact structures through a coarsening process constrained by the growing gel network. The dynamics transition from diffusive to superdiffusive behavior indicates the arrest of the phase-separated state and formation of the macroporous structure. Higher PEO content induces earlier gelation and, consequently, earlier arrest of the phase-separated structure, leading to smaller macropores. Although the initial stages of gelation and phase separation could not be captured, the subsequent coarsening process was clearly resolved, providing valuable insight into structural evolution in later stages. Palladium interacts mainly with PEO and does not affect the structural and dynamic evolution during gelation. After calcination, the palladium nanoparticles partially fill the mesopores. Complex macropore formation through sol–gel combination and phase separation was revealed by combining structural and dynamic evolution, providing guidelines for the design of hierarchically porous silica with optimized properties for advanced applications. The early stages of phase separation remain unresolved because the fast dynamics occur beyond the experimentally accessible time window. Detectors with higher frame rates, capable of capturing submillisecond dynamics, can probe the initial regime. Our study paves the way for future experiments to investigate the early stages of gelation and phase separation by combining fast XPCS and USAXS. As an established technique for investigating colloidal gels, XPCS combined with USAXS has great potential to explore a variety of gelation systems, which can significantly advance the understanding and control of properties in diverse soft matter systems.

## Supplementary Material



## Notes

Thermal analysis results, STEM images analysis, USAXS profiles for determination of radiation damage, parameters obtained from SAXS curve modeling and corresponding plots, intensity autocorrelation functions for SiPEO0 sample, *q*-dependence of relaxation rate and gamma for SiPEO6 sample, time evolution of the relaxation time, and analysis of anisotropic dynamics (PDF)
