# Supplementary material for: In Situ Coherent X‑Ray Scattering Investigation of Macropore Formation in Porous Silica
Source: ACS Omega. 2026 Mar 5;11(10):15820–9. doi: 10.1021/acsomega.5c08905 (PMC13000594; doi:10.1021/acsomega.5c08905)
Supplement: Supplementary file 1 [file ao5c08905_si_001.pdf]

Supporting Information

In situ coherent X-ray scattering investigation of macropore formation in porous silica

*Lucas A. Portela, Aline R. Passos**

AUTHOR ADDRESS

Lucas A. Portela - Brazilian Synchrotron Light Laboratory (LNLS), Brazilian Center for Research in Energy & Materials (CNPEM), Campinas, São Paulo 13083-970, Brazil

Aline R. Passos - Brazilian Synchrotron Light Laboratory (LNLS), Brazilian Center for Research in Energy & Materials (CNPEM), Campinas, São Paulo 13083-970, Brazil

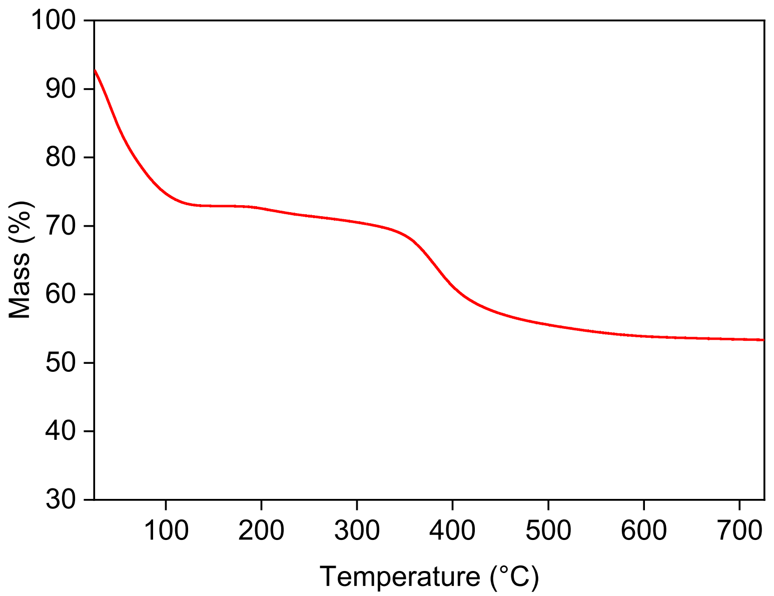

Figure S1. Thermogravimetric curve for the dried samples prepared with PEO.

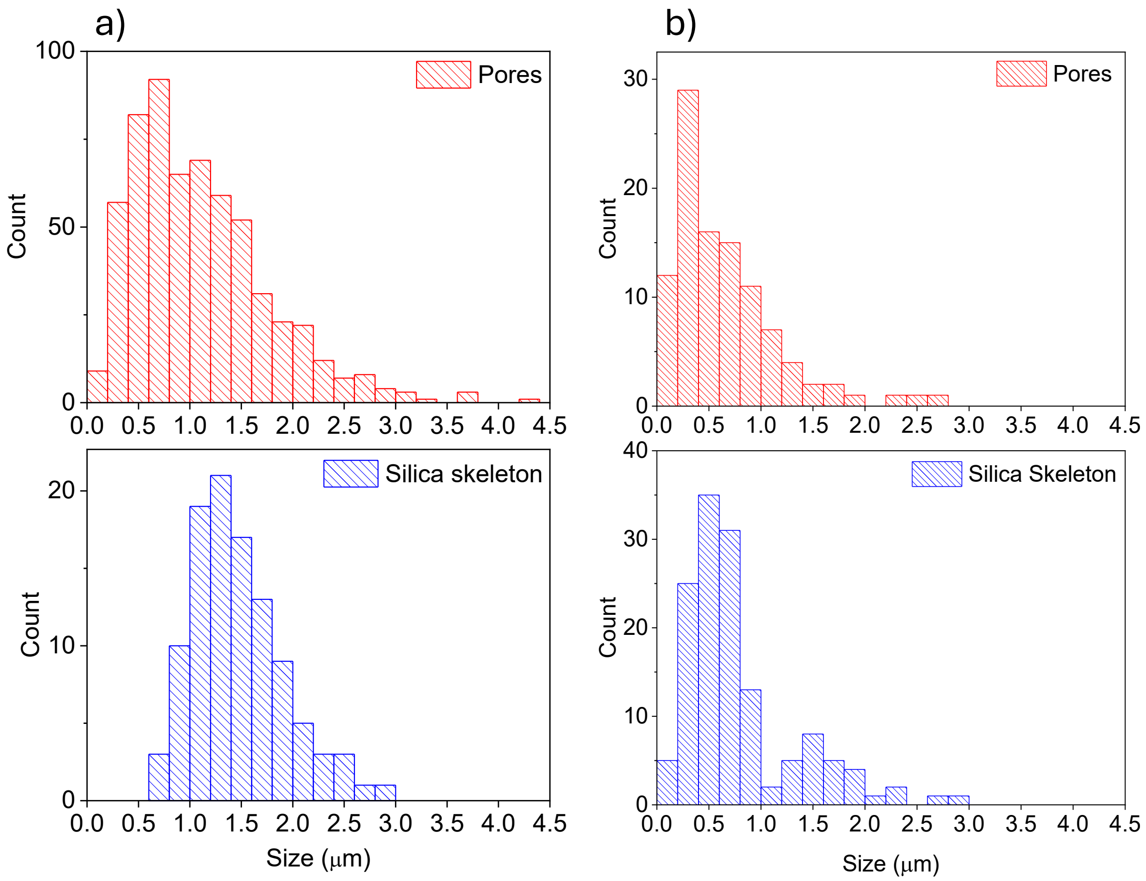

Figure S2. Size distribution of macropore diameter and silica skeleton thickness for the sample (a) SiPEO6 and (b) SiPEO7.

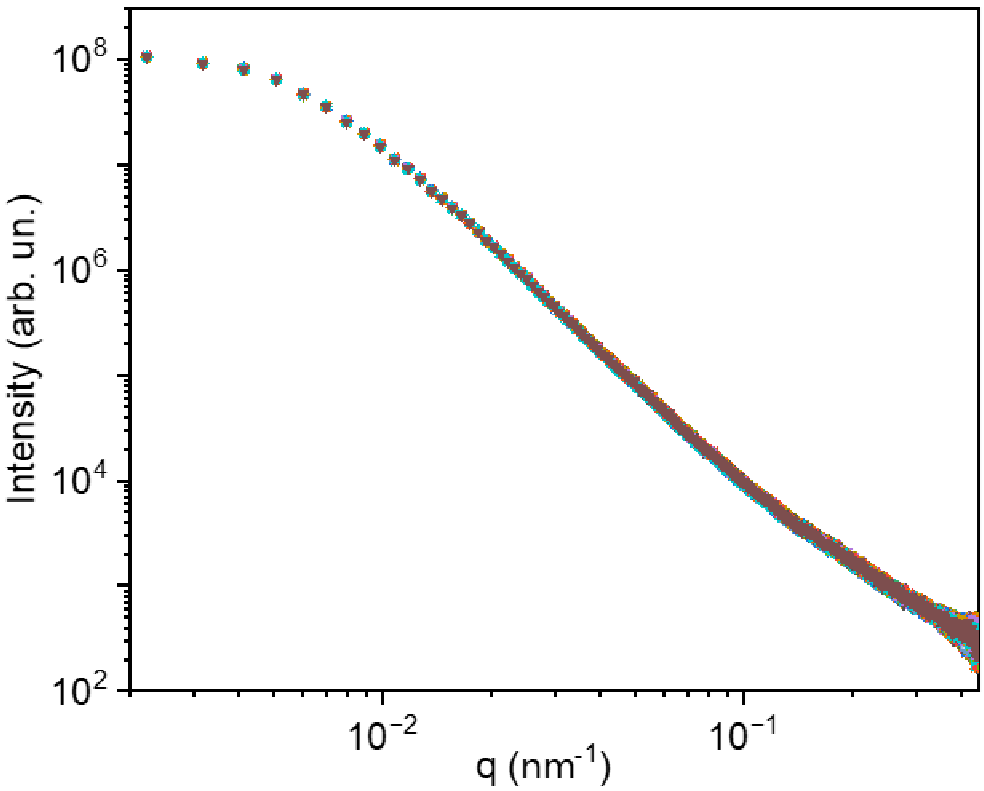

Figure S3. USAXS profiles obtained from the XPCS dataset, a sequence of 500 frames collected with 1s exposure time for the same spot on the sample SiPEO6 at 360 min.

Table S1. Guinier-Porod fitting parameters and results for the SiPEO6, SiPEO7 and PdSiPEO7 at different reaction time.

Sample	q-range (nm^{-1})	Rg (nm)	s	Porod expoent
SiPEO6-220min	0.006-0.48	$13 \pm 2 \times 10^{-4}$	$0.3 \pm 3 \times 10^{-6}$	$1.34 \pm 2 \times 10^{-7}$
SiPEO6-230min	0.006-0.48	$17 \pm 2 \times 10^{-4}$	$0.3 \pm 2 \times 10^{-6}$	$1.51 \pm 2 \times 10^{-7}$
SiPEO6-240min	0.002-0.48	$23 \pm 3 \times 10^{-4}$	$0.3 \pm 9 \times 10^{-7}$	$1.65 \pm 2 \times 10^{-7}$
SiPEO6-250min	0.002-0.48	$37 \pm 4 \times 10^{-4}$	$0.3 \pm 9 \times 10^{-7}$	$1.76 \pm 2 \times 10^{-7}$
SiPEO6-260min	0.002-0.48	$95 \pm 6 \times 10^{-4}$	$0.2 \pm 6 \times 10^{-7}$	$1.98 \pm 2 \times 10^{-7}$
SiPEO6-270min	0.002-0.48	$113 \pm 6 \times 10^{-4}$	$0.2 \pm 8 \times 10^{-7}$	$2.16 \pm 4 \times 10^{-7}$
SiPEO6-280min	0.002-0.48	$125 \pm 6 \times 10^{-4}$	$0.3 \pm 6 \times 10^{-7}$	$2.41 \pm 4 \times 10^{-7}$
SiPEO6-290min	0.002-0.48	$136 \pm 5 \times 10^{-4}$	$0.3 \pm 5 \times 10^{-7}$	$2.6 \pm 2 \times 10^{-7}$
SiPEO6-300min	0.002-0.48	$145 \pm 4 \times 10^{-4}$	$0.3 \pm 4 \times 10^{-6}$	$2.77 \pm 3 \times 10^{-7}$
SiPEO6-310min	0.002-0.48	$155 \pm 4 \times 10^{-4}$	$0.3 \pm 3 \times 10^{-6}$	$2.88 \pm 3 \times 10^{-7}$
SiPEO6-320min	0.002-0.48	$170 \pm 4 \times 10^{-4}$	$0.3 \pm 3 \times 10^{-6}$	$2.96 \pm 3 \times 10^{-7}$
SiPEO6-330min	0.002-0.48	$177 \pm 4 \times 10^{-4}$	$0.3 \pm 3 \times 10^{-6}$	$3.02 \pm 3 \times 10^{-7}$
SiPEO6-340min	0.002-0.48	$183 \pm 4 \times 10^{-4}$	$0.3 \pm 3 \times 10^{-6}$	$3.06 \pm 3 \times 10^{-7}$
SiPEO6-350min	0.002-0.48	$187 \pm 4 \times 10^{-4}$	$0.3 \pm 3 \times 10^{-6}$	$3.09 \pm 3 \times 10^{-7}$
SiPEO6-360min	0.002-0.48	$190 \pm 4 \times 10^{-4}$	$0.3 \pm 3 \times 10^{-6}$	$3.11 \pm 3 \times 10^{-7}$
SiPEO7-220min	0.006-0.48	$21 \pm 6 \times 10^{-4}$	$0.2 \pm 2 \times 10^{-6}$	$1.59 \pm 3 \times 10^{-6}$
SiPEO7-230min	0.006-0.48	$28 \pm 3 \times 10^{-4}$	$0.3 \pm 2 \times 10^{-6}$	$1.72 \pm 1 \times 10^{-6}$
SiPEO7-240min	0.002-0.48	$45 \pm 4 \times 10^{-4}$	$0.3 \pm 7 \times 10^{-7}$	$1.82 \pm 1 \times 10^{-7}$
SiPEO7-250min	0.002-0.48	$72 \pm 5 \times 10^{-4}$	$0.3 \pm 6 \times 10^{-7}$	$1.99 \pm 6 \times 10^{-7}$
SiPEO7-260min	0.002-0.48	$87 \pm 4 \times 10^{-4}$	$0.3 \pm 5 \times 10^{-7}$	$2.19 \pm 5 \times 10^{-7}$
SiPEO7-270min	0.002-0.48	$98 \pm 4 \times 10^{-4}$	$0.3 \pm 4 \times 10^{-7}$	$2.38 \pm 5 \times 10^{-7}$
SiPEO7-280min	0.002-0.48	$109 \pm 4 \times 10^{-4}$	$0.3 \pm 3 \times 10^{-7}$	$2.55 \pm 4 \times 10^{-7}$
SiPEO7-290min	0.002-0.48	$119 \pm 3 \times 10^{-4}$	$0.3 \pm 3 \times 10^{-7}$	$2.69 \pm 4 \times 10^{-7}$
SiPEO7-300min	0.002-0.48	$124 \pm 4 \times 10^{-4}$	$0.3 \pm 3 \times 10^{-7}$	$2.8 \pm 3 \times 10^{-7}$

SiPEO7-310min	0.002-0.48	$131 \pm 2 \times 10^{-4}$	$0.3 \pm 2 \times 10^{-7}$	$2.87 \pm 3 \times 10^{-7}$
SiPEO7-320min	0.002-0.48	$137 \pm 2 \times 10^{-4}$	$0.3 \pm 2 \times 10^{-7}$	$2.92 \pm 3 \times 10^{-7}$
SiPEO7-330min	0.002-0.48	$141 \pm 2 \times 10^{-4}$	$0.3 \pm 2 \times 10^{-7}$	$2.95 \pm 3 \times 10^{-7}$
SiPEO7-340min	0.002-0.48	$145 \pm 2 \times 10^{-4}$	$0.3 \pm 2 \times 10^{-7}$	$2.97 \pm 3 \times 10^{-7}$
SiPEO7-350min	0.002-0.48	$150 \pm 2 \times 10^{-4}$	$0.3 \pm 2 \times 10^{-7}$	$2.99 \pm 2 \times 10^{-7}$
SiPEO7-360min	0.002-0.48	$152 \pm 2 \times 10^{-4}$	$0.3 \pm 2 \times 10^{-7}$	$3.0 \pm 2 \times 10^{-7}$
PdSiPEO7-220min	0.009-0.48	$16 \pm 3 \times 10^{-4}$	$0.31 \pm 1 \times 10^{-6}$	$1.42 \pm 3 \times 10^{-6}$
PdSiPEO7-230min	0.009-0.48	$22 \pm 3 \times 10^{-4}$	$0.31 \pm 1 \times 10^{-6}$	$1.59 \pm 2 \times 10^{-6}$
PdSiPEO7-250min	0.006-0.48	$52 \pm 5 \times 10^{-4}$	$0.32 \pm 9 \times 10^{-7}$	$1.87 \pm 9 \times 10^{-7}$
PdSiPEO7-260min	0.002-0.48	$76 \pm 4 \times 10^{-4}$	$0.35 \pm 7 \times 10^{-7}$	$1.99 \pm 5 \times 10^{-7}$
PdSiPEO7-270min	0.002-0.48	$84 \pm 4 \times 10^{-4}$	$0.32 \pm 5 \times 10^{-7}$	$2.26 \pm 6 \times 10^{-7}$
PdSiPEO7-280min	0.002-0.48	$93 \pm 4 \times 10^{-4}$	$0.32 \pm 4 \times 10^{-7}$	$2.42 \pm 5 \times 10^{-7}$
PdSiPEO7-290min	0.002-0.48	$101 \pm 3 \times 10^{-4}$	$0.32 \pm 4 \times 10^{-7}$	$2.56 \pm 5 \times 10^{-7}$
PdSiPEO7-300min	0.002-0.48	$108 \pm 4 \times 10^{-4}$	$0.39 \pm 4 \times 10^{-7}$	$2.63 \pm 4 \times 10^{-7}$
PdSiPEO7-310min	0.002-0.48	$116 \pm 4 \times 10^{-4}$	$0.37 \pm 3 \times 10^{-7}$	$2.66 \pm 3 \times 10^{-7}$
PdSiPEO7-320min	0.002-0.48	$118 \pm 4 \times 10^{-4}$	$0.26 \pm 3 \times 10^{-7}$	$2.80 \pm 4 \times 10^{-7}$
PdSiPEO7-330min	0.002-0.48	$122 \pm 4 \times 10^{-4}$	$0.22 \pm 3 \times 10^{-7}$	$2.86 \pm 4 \times 10^{-7}$
PdSiPEO7-340min	0.002-0.48	$122 \pm 2 \times 10^{-4}$	$0.26 \pm 2 \times 10^{-7}$	$2.89 \pm 3 \times 10^{-7}$
PdSiPEO7-350min	0.002-0.48	$123 \pm 2 \times 10^{-4}$	$0.27 \pm 3 \times 10^{-7}$	$2.92 \pm 3 \times 10^{-7}$
PdSiPEO7-360min	0.002-0.48	$123 \pm 2 \times 10^{-4}$	$0.27 \pm 2 \times 10^{-7}$	$2.94 \pm 3 \times 10^{-7}$

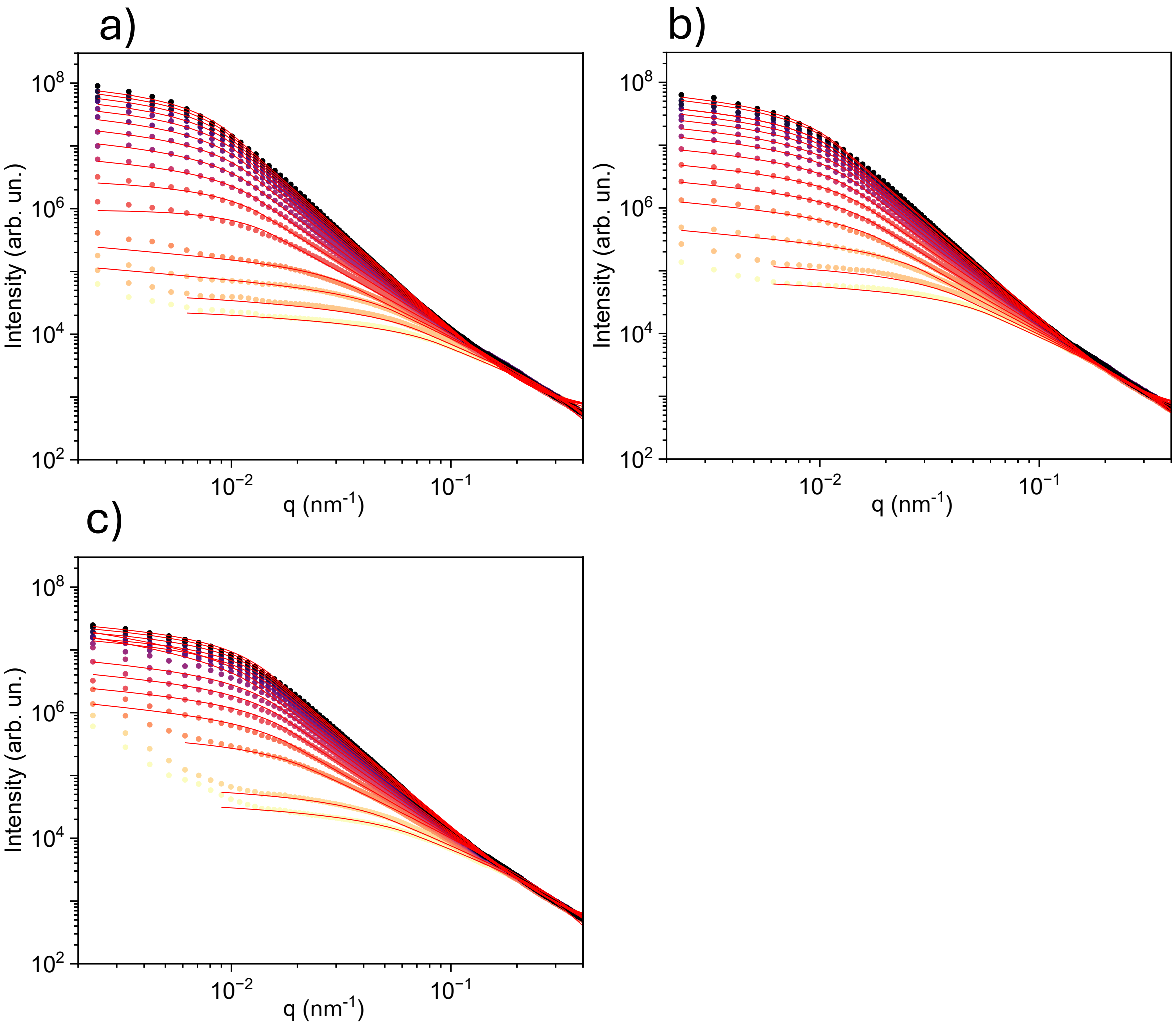

Figure S4. USAXS profiles during the gelation of (a) SiPEO6, (b) SiPEO7, and (c) PdSiPEO7 samples. The solid lines represent the fits using the Guinier-Porod model.

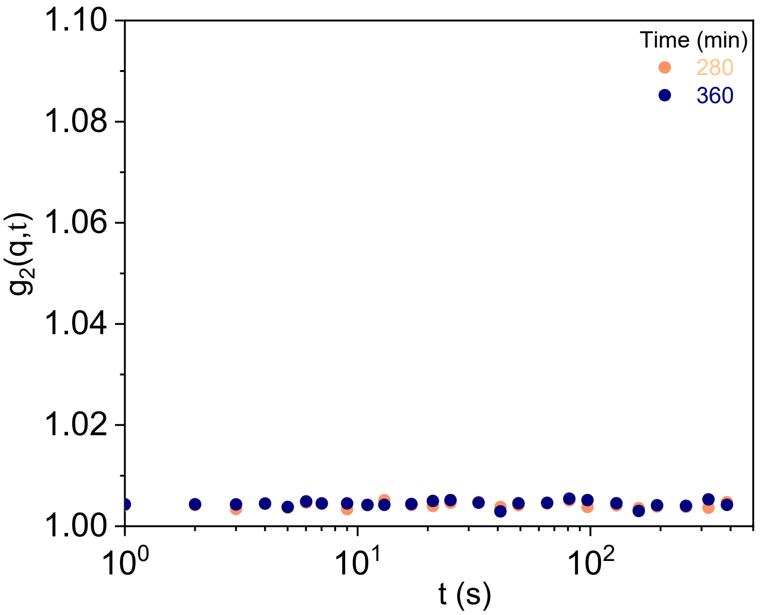

Figure S5. Intensity autocorrelation functions, $g_2(q, t)$ at $q = 0.0168\ nm^{-1}$ for the sample SiPEO0 at 280 and 360 min.

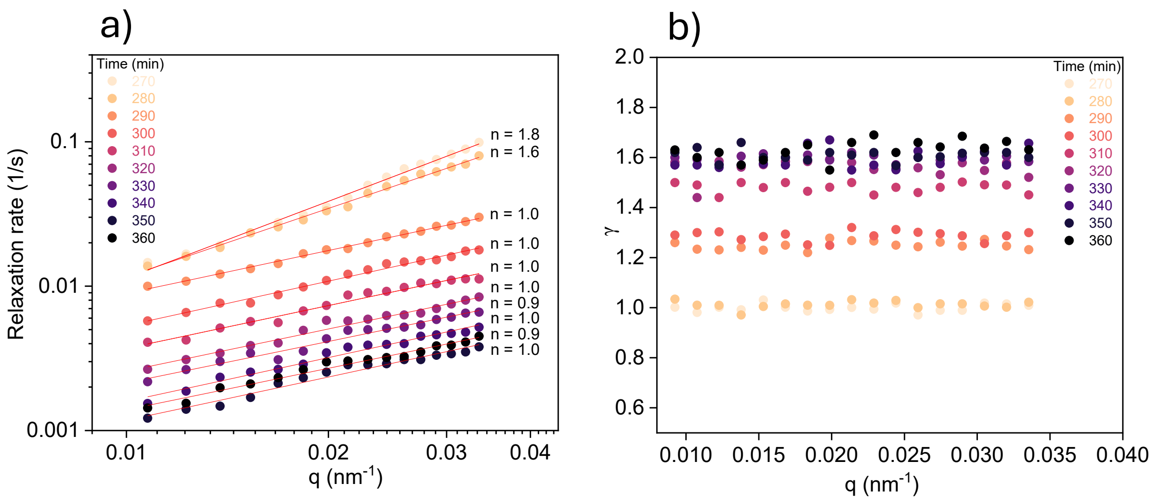

Figure S6. (a) q-dependence of relaxation rate (Γ), solid lines represent fits using the power-law $\Gamma = q^n$ and (b) gamma versus q for the sample SiPEO6.

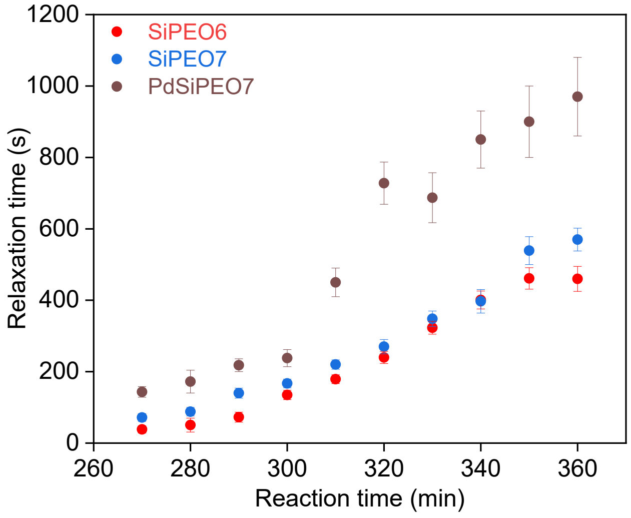

Figure S7. Time evolution of the relaxation time for the samples SiPEO6, SiPEO7 and PdSiPEO7.

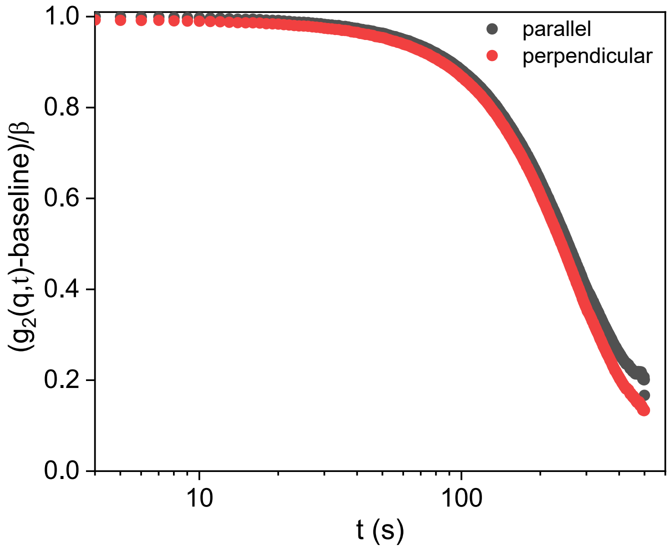

Figure S8. g_2 functions at q = 0.0168 nm^{-1} for the samples SiPEO6 at 360 min, calculated along directions parallel and perpendicular to the capillary sample holder, using an angular width of $\pi/6$.
